# Editorial: New insights on the relationship between neuroplasticity, genetic endophenotypes, and psychiatric disorders throughout aging and in the elderly population

**DOI:** 10.3389/fpsyt.2024.1375509

**Published:** 2024-02-16

**Authors:** Felipe Kenji Sudo, Viola Oertel, Sanjeev Kumar, Gilberto Sousa Alves

**Affiliations:** ^1^ D’Or Institute for Research and Education (IDOR), Rio de Janeiro, Brazil; ^2^ Laboratory for Neuroimaging, Department of Psychiatry, Psychosomatic Medicine and Psychotherapy, Goethe University, Frankfurt, Germany; ^3^ Centre for Addiction and Mental Health, Toronto, ON, Canada; ^4^ Temerty Faculty of Medicine, University of Toronto, Toronto, ON, Canada; ^5^ Translational Psychiatry Research Group, Federal University of Maranhão, São Luís, Brazil; ^6^ Post-Graduation in Psychiatry and Mental Health (PROPSAM), Federal University of Rio de Janeiro, Rio de Janeiro, Brazil

**Keywords:** geriatric psychiatry, aging, cognition, allostatic load (AL), stress, cognitive training, executive function, nasolacrimal canal obstruction

Beyond genetic predispositions, the notion that people’s illness profile and clinical outcomes may be driven by the degree of stress throughout their lifespan has become increasingly accepted. Allostatic load (AL), the wear and tear on organic systems due to chronic overactivity or inactivity of physiological processes in response to stress, has emerged as a popular concept to explain different phenotypes within groups with similar chronological ages (1). High AL has been linked to an array of clinical consequences consistently associated with aging, including metabolic diseases, neurodegenerative conditions, mood disorders, and mortality (2). However, the mechanisms underpinning the relationships between age-related changes and stress are still matter of debate. In this Research Topic, authors sought to explore these shortcomings through different approaches.

For instance, de Oliveira et al. investigated the impact of aging on neurophysiological reactions to acute stress, namely the startle reflex. In normal conditions, the startle reflex is reduced when the pulse is preceded by a weaker sensory stimulus (prepulse), characterizing the physiological phenomenon named “prepulse inhibition” (PPI). Previous evidence suggested that aging is associated with decreased startle reflex and increased startle latency (3). Moreover, a U-shaped function relationship was found between PPI and age (3). Given that other data indicated a positive correlation between PPI and cognitive performance in young adults (4), the authors hypothesized that PPI alterations could function as a biomarker of cognitive decline in normal aging. The results demonstrated a reduced PPI in healthy older subjects (n=14) in comparison with a younger group (n=14). Although no significant correlation was detected between PPI and scores in neuropsychological tasks in older participants, the study provides new insights into the aging-related brain changes affecting the sensorimotor functional integration.

Other studies addressed the issue of cognitive changes in older subjects by analyzing the relationships across mood disorders, executive function deficits, and markers of neural dysfunction. Chu et al. conducted a cross-sectional design in subjects with late-life depression (LLD, n=50) and Alzheimer’s disease (AD, n=50) and showed greater executive dysfunction in AD than in LLD, and greater accuracy of Trail Making Test A and B and Medial Temporal Atrophy (MTA) measurements to discriminate between these clinical groups. The study emphasizes the relevance of employing standard cognitive assessment tools to screen and detect dementia, which may be useful in the context of less economic resources, where advanced (CSF, blood) biomarkers are of high cost or not available.

As for Ma et al., the topic was approached through a pilot randomized trial in a community of 28 individuals in Hong Kong investigating the impact of computerized cognitive training (CCT) on executive dysfunction of LLD subjects. The intervention included 2 sessions per week, one hour each, along 6 weeks and the experimental group reached significant improvement in global cognitive function assessed using Montreal Cognitive Assessment (MoCA). Correlations between Hamilton depression scale improvement and increase in BDNF levels were also reported. Although results are limited by its reduced sample size and small statistical power, findings may encourage upcoming investigations on the effect of CCT on mood and BDNF, possibly establishing this intervention as effective for cognition in LLD.

Finally, stress related to medical conditions may also exert significant influence on mood states. In this perspective, Guo et al. analyzed the prevalence of depression and anxiety in adults who underwent dacryocystorhinostomy (DCR) due to nasolacrimal duct obstruction (NLDO). This condition can cause epiphora, blurred vision, and dry eye, which have been related to mental health disorders. Consistently, the authors found a positive association among dry eye, anxiety, and depressive symptoms.

The articles presented in this Research Topic may add to our understanding on how cognitive and mood disorders through the life cycle could be prevented, diagnosed, or treated. Future studies about the role of stress and AL on age-related neural changes ought to be ignited by this evidence, providing important advances in the knowledge of the processes implicated in late life mental health disorders.

## Author contributions

FS: Conceptualization, Data curation, Formal analysis, Funding acquisition, Investigation, Methodology, Project administration, Resources, Software, Supervision, Validation, Visualization, Writing – original draft, Writing – review & editing. VO: Conceptualization, Resources, Supervision, Validation, Visualization, Writing – original draft, Writing – review & editing. SK: Conceptualization, Resources, Supervision, Validation, Visualization, Writing – original draft, Writing – review & editing. GA: Conceptualization, Formal analysis, Funding acquisition, Investigation, Resources, Supervision, Validation, Visualization, Writing – original draft, Writing – review & editing.
